# Deep generative models of LDLR protein structure to predict variant pathogenicity

**DOI:** 10.1016/j.jlr.2023.100455

**Published:** 2023-10-11

**Authors:** Jose K. James, Kristjan Norland, Angad S. Johar, Iftikhar J. Kullo

**Affiliations:** 1 Department of Cardiovascular Medicine, Mayo Clinic, Rochester, MN, USA; 2 Gonda Vascular Center, Mayo Clinic, Rochester, MN, USA

**Keywords:** genomics, proteomics, physical biochemistry, dyslipidemias, lipoproteins/receptors, atherosclerosis

## Abstract

The complex structure and function of low density lipoprotein receptor (LDLR) makes classification of protein-coding missense variants challenging. Deep generative models, including Evolutionary model of Variant Effect (EVE), Evolutionary Scale Modeling (ESM), and AlphaFold 2 (AF2), have enabled significant progress in the prediction of protein structure and function. ESM and EVE directly estimate the likelihood of a variant sequence but are purely data-driven and challenging to interpret. AF2 predicts LDLR structures, but variant effects are explicitly modeled by estimating changes in stability. We tested the effectiveness of these models for predicting variant pathogenicity compared to established methods. AF2 produced two distinct conformations based on a novel hinge mechanism. Within ESM’s hidden space, benign and pathogenic variants had different distributions. In EVE, these distributions were similar. EVE and ESM were comparable to Polyphen-2, SIFT, REVEL, and Primate AI for predicting binary classifications in ClinVar. However, they were more strongly correlated with experimental measures of LDL uptake. AF2 poorly performed in these tasks. Using the UK Biobank to compare association with clinical phenotypes, ESM and EVE were more strongly associated with serum LDL-C than Polyphen-2. ESM was able to identify variants with more extreme LDL-C levels than EVE and had a significantly stronger association with atherosclerotic cardiovascular disease. In conclusion, AF2 predicted LDLR structures do not accurately model variant pathogenicity. ESM and EVE are competitive with prior scoring methods for prediction based on binary classifications in ClinVar but are superior based on correlations with experimental assays and clinical phenotypes.

Familial hypercholesterolemia (FH) is vastly underdiagnosed (1), motivating a ‘genome first’ approach to detect cases (2). Pathogenic variants in low density lipoprotein receptor (*LDLR)* cause the majority of FH cases (>80% of molecularly defined cases) (3). The gene is listed as medically actionable by the American College of Medical Genetics and Genomics (4) leading to inclusion in commercial and direct to consumer sequencing panels. Classification of identified variants is complicated by discordance between submitters (5). A consensus guideline from the Clinical Genome Resource FH Variant Curation Expert Panel attempted to harmonize variant classification based on multiple lines of evidence (3). However, many variants are classified as variants of uncertain significance (VUS) often due to lack of functional data (6). As a result, novel predictive methods that improve the accuracy of variant classification are needed.

Experimental assays have been developed to assess LDLR surface expression, LDL uptake, and receptor recycling (7). However, the infrastructure and expertise required for these methods are barriers to implementation. On the other hand, in silico prediction tools are facile and ubiquitous. Experimental assays offer a more accurate identification of pathogenic variants and in one study improved the odds ratio (OR) for early myocardial infarction (MI) from 2.1 to 20 (8). The discrepancy between computational and experimental annotation of pathogenic variants highlights the lack of sensitivity and specificity of existing computational models for clinical phenotypes (9).

Common bioinformatic tools train supervised machine learning models on large datasets such as ClinVar to assign labels (10, 11). However, datasets like ClinVar exhibit significant bias because they involve the nonrandom selection of variants, an uneven distribution between pathogenic and benign labels, and a substantial proportion of variants have uncertain or conflicting classifications (12). Moreover, it is difficult to know which variants were used for training, making data leakage a significant issue when assessing model performance (13). These biases may inflate the predictive capacity of supervised learning methods and decrease generalizability.

Evolutionary models rely on the principle that the structure and function of a protein is encoded within a amino acid sequence whose salient features can be determined from a diverse set of sequences for similar functioning proteins. At the most basic level, evolutionary models can represent a substitution matrix where scores are based on amino acid similarity (14). Methods like SIFT improve accuracy by including positional conservation with amino acid propensity to determine the probability of amino acid substitution (15). However more recent methods leverage covariation between residues in a multiple sequence alignment (MSA) to estimate epistatic interactions (16).

Learning models for variant prediction can be classified as “generative” when they estimate a probability distribution, P(S), for a given sequence S. Initial generative models for sequence prediction were statistical models that used pairwise correlations between residues to calculate a log likelihood score comparing variant sequence to wild type (17). Including higher order interactions within these models required estimating several orders of magnitude more parameters and was computationally intractable. Deep learning models, which are neural networks that consists of multiple hidden layers, were introduced as a solution to capture these higher order interactions without requiring explicit representations (18). These models create representations of a protein sequence within their hidden space that correlate with properties relevant for protein evolution, structure, and fitness (19).

We introduce two deep generative models with different model architectures and training methods that are used for variant effect prediction. The evolutionary model of variant effect (EVE) model trains a variational autoencoder (VAE) from a MSA (20). Evolutionary scale modeling (ESM) on the other hand trains a transformer using an unaligned global protein dataset consisting of millions of diverse sequences (21, 22). It is important to recognize that these models are not explicitly trained to predict variant effects but are used for prediction by means of “transfer learning”. This is a learning strategy where models are trained on a task where there is an abundance of data such as creating a general representation of protein sequences from unlabeled sequences and then applied to tasks with limited data availability such as variant effects and protein structure. Despite this unsupervised training strategy, both ESM and EVE are highly predictive of disease causing variants compared to supervised methods (23, 24).

ESM and EVE are exclusively data-driven models and do not incorporate biophysical constraints for prediction. As a result, they are able to build complex data representations of epistatic interactions but are also difficult to interpret without adding additional constraints on the network (18). In contrast, models with strong biological priors, such as physics-based models from protein structures, offer more mechanistic insight of variant effects but may be limited to single conformations (25). Transformer architectures have been utilized for structure prediction and demonstrated using ESM (21). More recently AlphaFold 2 (AF2) was developed and shown to accurately model diverse structures across the proteome (26). While AF2 was not designed to predict the effects of variants (https://alphafold.ebi.ac.uk/faq), the use of AF2 structures for variant prediction is an active area of research with the expectation that improved structural modeling of the human proteome may provide insight toward the mechanism of disease-associated variants (27). Recently, AF2 has been used to model pathogenic variants in *LDLR* (28) and *PCSK9* (29) to determine effects on protein-protein interactions and the catalytic domain, respectively. One approach for variant prediction is to directly model a variant sequence with AF2 and observe any conformational changes in the predicted structure or model confidence. However, this approach does not significantly alter predicted conformations from wild type, and changes in model confidence do not correlate with thermodynamic stability or protein function (30, 31). Another approach is to take the predicted wild-type structure and use structure-based forcefields to model variants and estimate changes in thermodynamic stability from the original wild-type structure (32). This latter approach is generally practiced (32) and what we have adopted to predict effects of *LDLR* variants.

Since generative models create representations of epistatic relationships within protein sequences from evolutionary history, we hypothesized that they better reflect a range of phenotypic effects than models which rely on supervised training. We tested our hypothesis using models that are distinct in their inputs, outputs, and architecture. Both ESM and EVE directly estimate the likelihood of a variant sequence based on evolutionary history but differ in training and overall architecture. AF2 uses a MSA as input but has a unique architecture and ultimately predicts a protein structure. These three models were compared to established bioinformatic scoring systems that range in complexity from relying solely on changes in amino acid chemistry (e.g., BLOSUM62) (33) and positional conservation (e.g., SIFT) (34) to supervised machine learning (e.g. Polyphen-2) (11), semi-supervised deep learning (e.g., Primate AI) (35), and meta-predictors (e.g., REVEL) (10). Our study used multiple modalities to test variant phenotype, including variant classification in the ClinVar database, LDL-C uptake of variants from a previously published experimental assay, and associations with serum LDL-C levels and atherosclerotic cardiovascular disease (ASCVD) outcomes from the UK Biobank (UKB).

## Materials and methods

### Generative deep learning models

#### AlphaFold 2

AF2 uses a novel transformer architecture to predict protein structures from sequence (26). The model utilizes two inputs, a MSA and a set of templates, or experimentally derived protein structures thought to be similar based on the input sequence. AF2 is a complex model incorporating multiple specialized transformers to generate a protein structure from these inputs. The first series of transformers create representations from pairwise correlations between residues using a MSA and refines distances in the pairwise model by enforcing geometric constraints. These representations are inputted into a second series of specialized transformers that yields an explicit structure. Training compares predicted structures with experimental structures using multiple scoring functions.

For our application of AF2, source code for release v2.2.4 was downloaded online and installed using the provided instructions (26). Input for the run script includes the FASTA protein sequence of human LDLR isoform1, which was obtained from Uniprot (P01130-1) (36), and a max template date set to 2022-01-10. A total of five structures were produced, and each is associated with an overall and per-residue predicted local distance difference test (pLDDT) score, which is a provided confidence metric that AF2 outputs. The pLDDT score predicts the agreement between a generated and experimental structure based on the local distance difference test (37). AF2 estimates the pLDDT using a supervised neural network that was trained on known experimental structures.

The top two structures by pLDDT score were obtained for further refinement. Calcium ions were added to LDLR type A (LA) repeats 1–7 and epidermal growth factor (EGF) A and B manually using PyMol (38) by aligning representative crystal structures of each domain. Side chain rotamers were relaxed while keeping the backbone fixed using the relax.linuxgccrelease function in Rosetta (39) resulting in five optimized structures. The most favorable scored structure was inputted into MutateX (40), which calculates changes in the Gibbs free energy of folding (ΔΔG) from five iterations of mutagenesis using the FoldX forcefield (41).

#### EVE

EVE uses a VAE to estimate a probability distribution of homologous sequences for a specific protein (20). The model assumes that a hidden space, also called a latent space (z), exists from which we can estimate the marginal probability of a given protein sequence (s) from trained model parameters (θ).

(1) p(s|θ)=∫p(s,z|θ)dz

The hidden space can be viewed as a compressed representation of salient features required for determining protein sequence. However, explicit derivation of this integral is high dimensional and computationally intractable. Instead, EVE utilizes a VAE which approximates the marginal probability, p(s|θ), for a given sequence. Use of VAEs for variant prediction was first proposed by DeepSequence (18), which EVE further improves. In general, the VAE architecture consists of an encoder neural network that reduces a high-dimensional input, such as a protein sequence, to a low dimensional hidden space and then recreates the protein sequence with a decoder neural network. The encoder estimates a posterior distribution, $q\left(z|s,\theta_{encoder}\right)$, from a protein’s MSA. The decoder generates protein sequences by estimating the likelihood of a sequence, $p\left(s|\;z,\theta_{decoder}\right)$, using a sampled prior distribution, p(z). The prior distribution is assumed to be Gaussian for simplicity. An Evidence of Lower Bound (ELBO) function estimates the lower bound of the marginal probability for a sequence from the expected log-likelihood of a predicted sequence and the Kullback–Leibler divergence ($D_{KL}$) between the posterior and prior distributions.

(2) $$\log\;p\left(s|\theta \right)\geq ELBO\left(s\right)=E_{q}\left[\log\;p\left(s|z,\theta_{decoder}\right)\right]-D_{KL}\;\left(q\left(z|s,\theta_{encoder}\right)\|p\left(z\right)\right)$$

Training the VAE maximizes the ELBO function. The negative log likelihood of a given variant sequence with respect to the wild-type sequence can be calculated as the difference between their respective ELBO functions.

(3) $$-\log\frac{p\left(s_{variant}|\theta \right)}{p\left(s_{wildtype}|\theta \right)}\;∼\;ELBO\left(s_{wildtype}\right)-ELBO\left(s_{variant}\right)$$

In the EVE approach, the negative log likelihoods are referred to as the evolutionary index. EVE scores utilize a Gaussian mixture model on the evolutionary index to standardize it, ensuring that the resulting scores fall within a range of 0–1. To obtain EVE scores for our study, the original MSA for training was obtained from the Marks’ lab sever (evemodel.org/proteins/LDLR_HUMAN), and available source code was used to calculate EVE scores with default parameters unless otherwise specified in the text.

#### ESM

ESM is a transformer model related to the family of Bidirectional Encoder Representations from Transformers (BERT) language models (21). Inputs in the model are protein sequences, with each amino acid represented by individual tokens. The transformer neural architecture uses self-attention to explicitly assess pairwise correlations between all tokens in a sequence. Each sequence additionally has a classification token which is further processed to a pooled output vector and can be considered as a hidden representation of the entire sequence. The model is trained using a masked language model, where the probability for a sequence (s) is calculated as the product of the conditional probabilities for individual amino acids at each residue ($s_{i}$) given the surrounding sequence.

(4) $$p\left(s\right)=\prod_{i=1}^{n}p\left(s_{i}|s_{1},s_{2}\ldots \;s_{i-1},s_{i+1}\ldots \;s_{n-1},s_{n}\right)$$

The training objective is the average cross entropy loss between the model’s predictions and the true token from masked residues. Variant effects can be predicted by an estimation of the log-likelihood of the variant sequence compared to the wild-type.

We used a version of ESM, ESM-1v from Meier *et al.* (22), intended for variant prediction. The model utilizes 650 million parameters and was trained with sequences from the UniRef90 dataset. A total of five models of ESM1-v were produced that were initialized with different seeds. Each model was downloaded, and the human LDLR isoform 1 protein sequence was used as input to calculate log-likelihood scores for all possible variants. Of note, this model does not require MSA. To obtain the hidden space representation and final score for each variant, the outputs from all five models were averaged.

### ClinVar *LDLR* variant classifications

The ClinVar dataset was obtained from a public archive (42) in October 2022. Variants with at least a one-star rating were selected. ClinVar classifications include benign, likely benign, VUS, conflicting interpretation, likely pathogenic, and pathogenic. Classifications were further simplified such that likely benign and benign were labeled as benign, and likely pathogenic and pathogenic were labeled as pathogenic. REVEL, Polyphen-2-HDIV, and Primate AI scores were obtained for all variants in the dataset using SnpEff (43). BLOSUM62 scores were manually calculated based on the publish matrix (44). Scores were further curated to eliminate values assigned to alternative isoforms. EVE scores, ESM scores, and ΔΔG values from AF2 models were additionally added to the dataset.

### Burden testing

For *LDLR* rare variant burden association testing, we used the UK Biobank 500k exome sequencing dataset provided on DNAnexus. We included 448,535 individuals of European ancestry, determined by supervised ADMIXTURE analysis, with K = 5, representing the five superpopulations from the 1,000 Genomes dataset that we used as our reference panel. We used Regenie v3.2.6 to perform the burden association testing between missense variants in *LDLR* and the phenotypes (LDL-C and ASCVD subtypes). We restricted the analyses to variants in *LDLR* with allele frequency <0.1%. We used leave-one out cross validation. In step 1, we used array genotyping data, filtered with plink using ‘maf 0.01 --mac 100 --geno 0.1 --hwe 1e-15 --mind 0.1’. In step 2, we used the fast Firth correction as fallback for *P*-values below 0.05. We adjusted for age, sex, and 20 principal components. We transformed LDL-C using rank-based inverse normal transformation. To define the ASCVD phenotypes, we used ICD codes, CPT codes, self-reported data, and the algorithmically defined outcomes for MI and stroke generated by the UK Biobank.

### Statistical analysis

Receiver operative curves and area under the curve calculations were calculated using the pROC R package with confidence intervals calculated using stratified bootstrapping (45). Visualization of high dimensional representations for sequences across two dimensions was accomplished using the python UMAP package (46) with parameters n_neighbors set to 40 and min_dist set to 0.1.

## Results

### AF2 generates two major conformations for LDLR

**Fig. 1:**
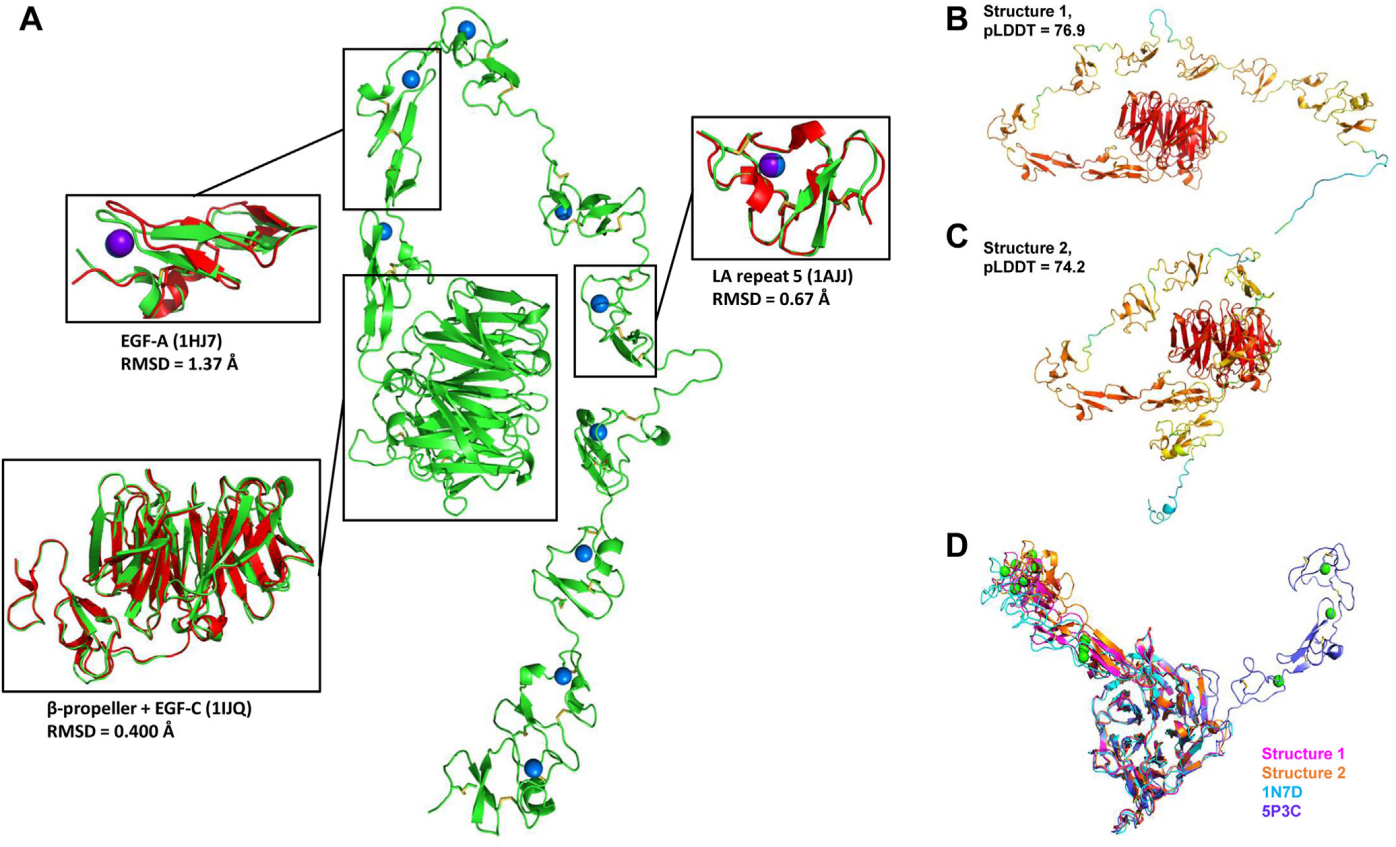
**Predicted LDLR structures from AlphaFold2.** (A): The predicted extracellular region of LDLR is shown (green) and compared against experimental structures (red, PDB ID within parenthesis) of individual domains. Calcium ions are added as blue within the AF2 structure and purple within experimental structures. Disulfide bonds are in yellow. The two major predicted conformations are shown as (B) structure 1 and (C) structure 2, and colorized by per-residue pLDDT score. The overall pLDDT score for the structure is shown. (D): Orientation between the EGF-A and EGF-B repeats from the beta-propeller domain is compared between predicted structures and crystal structures in neutral (PDB ID: 3P5B) and acidic (PDB ID: 1N7D) conditions.

AF2 structures were generated from the canonical LDLR isoform 1 amino acid sequence, which was used to create a MSA consisting of 15,161 sequences. A total of five structures were produced (supplemental Fig. S1). Model confidence was quantified using pLDDT by residue and for the entire structure. AF2 accurately models domains with experimental structures (Fig. 1A). The LDLR extracellular structure contains two main domains, the ligand-binding domain which consists of seven LA repeats in series and a EGF precursor homology domain that consists of two EGF domains (EGF-A and EGF-B) followed by a beta-propeller domain and a final EGF-C domain (47). The LA repeats bind calcium and are responsible for interaction with lipoproteins (48). Compared to the crystal structure, AF2 was able to reproduce observed disulfide bonds and coordination of the metal ion without explicitly modeling the calcium ion. The EGF precursor homology domain is responsible for large-scale conformational changes that regulates binding and release of the lipoprotein cargo in endosomal pH (49). AF2 structures were able to accurately represent both the EGF and beta-propeller domains. The remaining C-terminal region in the AF2 structures consists of a largely disordered region rich in serine and threonine residues that undergo O-linked glycosylation followed by a α-helical transmembrane region and disordered intracellular region. There are no experimental structures for these regions; however, the expected oligosaccharide posttranslational modifications are not modeled in the AF2 structures.

Two global conformations were predicted from AF2 depicting an extended conformation between LA repeats (structure 1, Fig. 1B) and a kinked conformation (structure 2, Fig. 1C). Structure 1 produced multiple interactions between LA repeats 4 and 5 and the beta-propeller domain (supplemental Fig. S2A). Structure 2 produced multiple interactions between LA repeats 2–6 and the beta-propeller (supplemental Fig. S2B). Predicted structures showed an unstructured linker region (res. 185–196) between LA repeats 4 and 5 that acts like hinge orienting LA repeats 1–4 in an extended or kinked conformation. The hinge region had an average pLDDT score of 46.17 in structure 1 and 44.51 in structure 2, indicating poor model confidence and intrinsic disorder (50).

Previously published experimental structures suggested a hinge region exists between the EGF-B and beta-propeller domains where a conformational switch was hypothesized to regulate an open conformation in extracellular pH and closed conformation in endosomal pH (51). A rotation between these two domains was observed from crystal structures at neutral and acidic pH (52, 53). However, predicted structures from AF2 generally agree on a single conformation at this region with an orientation similar to the experimental conformation at acidic pH (Fig. 1D). Residues 394–399 comprise the linker region separating EGF-B and the beta-propeller and had a mean pLDDT in both predicted structures of 83.36. This suggested good model confidence but below the generally accepted high accuracy cut-off of 90.

Variant effects were estimated by calculating changes in the Gibbs free energy of folding (ΔΔG) by using the FoldX forcefield after mutagenesis on the wild-type structure (54). Most variants yielded a ΔΔG value near or greater than 0. Values greater than zero suggest a destabilizing effect on the structure, whereas values below zero suggest a stabilizing effect. Since either case may lead to disease, using the absolute free energy change (|ΔΔG|) improves performance for identifying pathogenic variant effects (25). Calculated |ΔΔG| values between structures 1 and 2 showed agreement (Spearman correlation coefficient, ρ = 0.65 and *P*-value < 0.0001, supplemental Fig. S3). Disagreement between models was greater with larger |ΔΔG| values, corroborating observations that conformational differences in the two structures are structurally significant.

### ESM clusters LDLR variants by pathogenicity

**Fig. 2:**
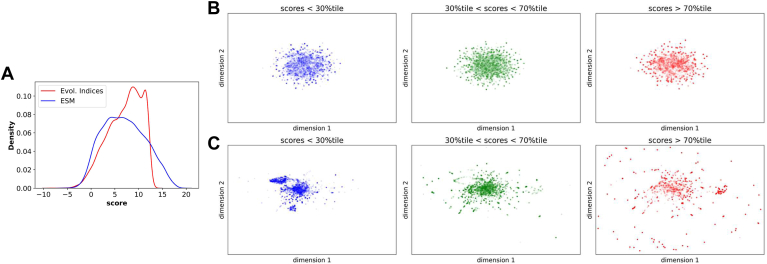
**Distribution of variant sequencesin EVE and ESM.** Both ESM scores and the precursor to EVE scores, referred to as evolutionary indices, estimate the log-likelihood ratio of a variant sequence compared to the wild type. (A): The distribution of evolutionary indices (red) and ESM scores (blue) across all shared variants is shown. Each variant sequence is also represented within the hidden space of (B) EVE and (C) ESM. Dimensionality reduction for visualization was performed with UMAP. Panels contain variants that are categorized by their percentile ranks as labeled.

EVE scores were trained on the original 4,048 sequence MSA used by the Marks lab yielding scores for all possible 14,997 variants covering 88.7% of residues. ESM was already pretrained, and scores were obtained for all possible 16,340 variants covering 100% of residues. The evolutionary index, a precursor to the EVE score, was used to compare distribution of scores with ESM since both estimated the negative log-likelihood of a variant sequence compared to wild type (Fig. 2A). In this comparison, a higher score corresponds to a lower predicted likelihood for a variant sequence and increased evidence for pathogenicity. Among variants that are shared between ESM and EVE, the former had a broader distribution of likelihoods (25th, 50th, and 75th percentile for ESM 3.51, 6.74, 10.28 vs. EVE 4.54, 7.67, 9.96).

We next compared distributions between high and low scoring variants within the hidden space of ESM and EVE (Fig. 2B). Since this space is multidimensional, we used UMAP to reduce the space to two-dimensions for visualization. Variants with ESM scores in the lower 30th percentile were tightly clustered compared to those with ESM scores in the upper 30th percentile which had a significantly broader distribution (Wilcoxon rank-sum *P* value 2.11e-232 in dimension 1 and 1.41e-28 in dimension 2). Scores between the 30th and 70th percentile had a distribution that is in between. For EVE, there was no clear differences in distribution between scores within the bottom 30th percentile and the top 30th percentile (Wilcoxon rank-sum *P* value 0.61 in dimension 1 and 1.37e-04 in dimension 2). Given that VAEs are also used for dimensionality reduction and may uncover different patterns between variant sequences, we retrained an EVE model with a two-dimensional latent space (supplemental Fig. S4). Based on this representation, variants in the top 30th percentile were tightly clustered, while those in the bottom 30th percentile were more broadly distributed (Wilcoxon rank-sum *P* value 1.01e-11 in dimension 1 and 8.45e-72 in dimension 2).

### EVE and ESM predicts pathogenic variants across LDLR domains and correlates with LDL uptake

**Fig. 3:**
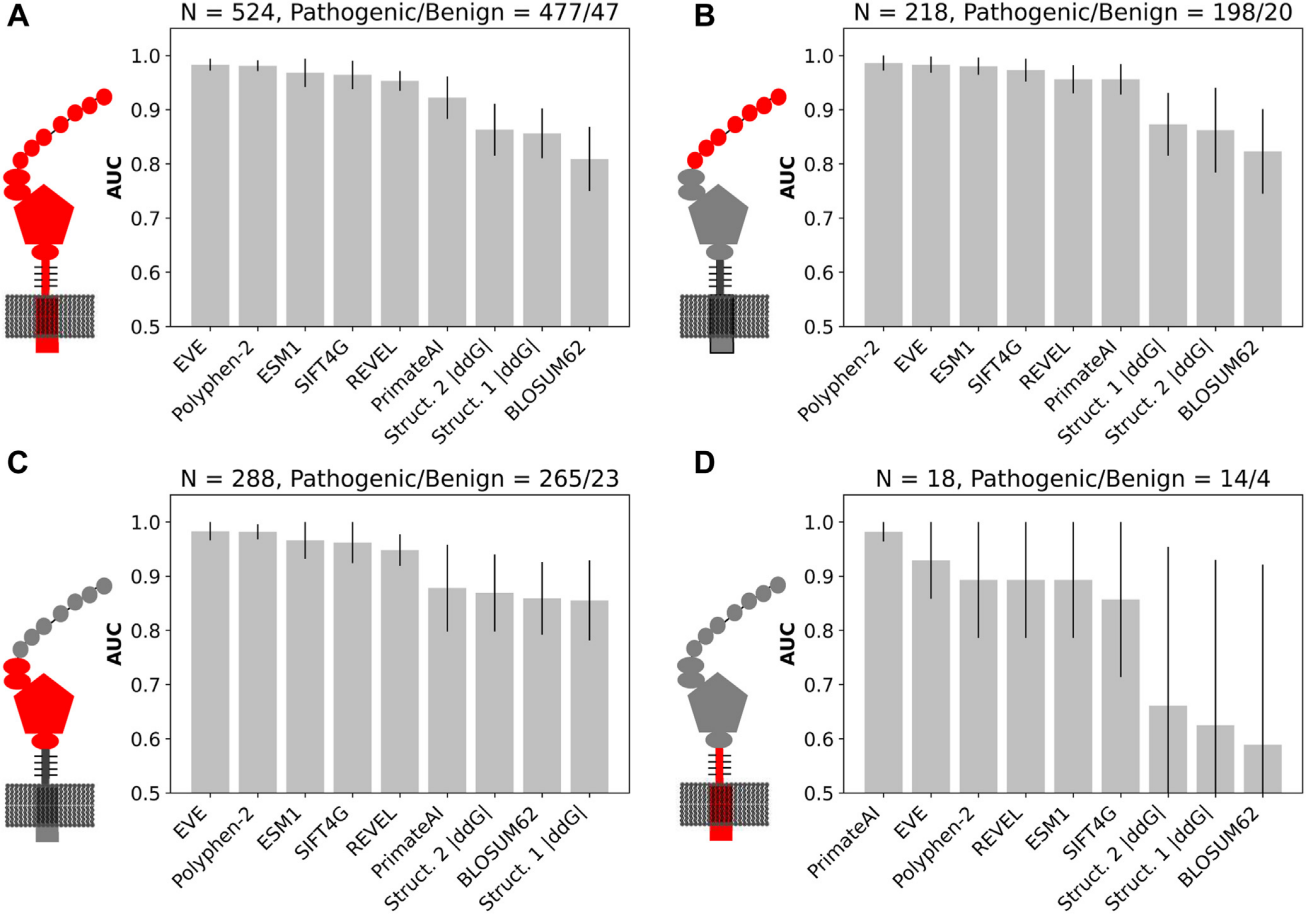
**Comparingpredictive scoresin ClinVar.** Variants were collected from ClinVar with a benign or pathogenic label and at least a one-star rating. Variant effect predictors were compared for the (A) entire LDLR protein. Prediction models were also compared by domains including the (B) ligand binding domain, (C) EGF precursor homology domain, and (D) C-terminal region. Model performance was determined by AUC, which is shown for each model in descending order. Titles above each plot displays the total number of variants (N) and the number of pathogenic and benign variants.

The performance of EVE, ESM, and |ΔΔG| values from AF2 structures were compared with SIFT, BLOSUM62, Primate AI, PolyPhen-2, and REVEL using area under the receptor operating characteristic curve (AUC) from labeled data in ClinVar (Fig. 3). Including; all residues, EVE had an AUC of 0.983 (95% CI 0.972–0.993). ESM had an AUC of 0.968 (95% CI 0.943–0.994). AF2 structures 1 and 2 had an AUC of 0.856 (95% CI 0.81–0.903) and 0.863 (95% CI 0.815–0.911), respectively. SIFT4G, Polyphen-2, REVEL, and Primate AI had AUCs of 0.964 (95% CI 0.938–0.989), 0.981 (95% CI 0.971–0.991), 0.953 (95% CI 0.935–0.971), and 0.922 (95% CI 0.883–0.961) respectively. BLOSUM62 had an AUC of 0.809 (95% CI 0.750–0.868). Next, performance was compared by regions comprising functional domains. All models showed improved or similar AUCs with variants in the ligand-binding domain. The EGF precursor domain in comparison had a modest decline in AUCs for most models. A greater decline in AUC was found for most models within the disordered C-terminal region except Primate AI, whose AUC improved above all (0.982, 95% CI 0.933–1.0). However, the C-terminal region had the fewest number of available variants in ClinVar. In general, EVE had the highest overall AUC but was very similar to Polphen-2. ESM had lower AUCs in all domains and performed similarly to SIFT. Both AF2 structures had a lower AUCs compared to EVE and ESM and generally performed similarly to BLOSUM62.

Functional assays for LDLR measure a range of effects on LDL-C uptake. We next tested correlation between predictive scores and a previously published LDL-C uptake assay for 78 variants by Thormaehlen *et al.* (8) (supplemental Fig. S5). In their assay, LDL-C uptake is a unitless measure of the intracellular DiI-LDL signal quantified from background-subtracted images that reflected distinct intracellular compartments resembling endosomes or lysosomes. Benign variants for LDLR function would have higher LDL-C uptake measured than those with deleterious effects. ESM and EVE had *r*^2^ values of 0.379 (*P* value 2.57E-9) and 0.345 (*P* value 4.65E-6). From AF2 structure 1 and 2, |ΔΔG| values a *r*^2^ values of 0.143 (*P* value 6.88E-4) and 0.101 (*P* value 4.76E-3). BLOSUM62, SIFT4G, Polyphen-2, Primate AI, and REVEL had *r*^2^ values of 0.032 (*P* value 1.19E-1), 0.117 (*P* value 9.86E-3), 0.110 (*P* value 1.25E-2), 0.234 (*P* value 1.58E-4), and 0.191 (*P* value 7.51E-4), respectively.

### Model uncertainty correlates with better predictions for EVE

EVE scores were filtered based on uncertainty, which ranged from 0 to 1 with higher values reflecting greater uncertainty. Decreasing the uncertainty threshold to below 0.6 reduced the total number of labeled variables and improved the AUC to 0.985 (supplemental Fig. S6A). Decreasing the uncertainty threshold to 0.3 further improved AUC to 0.996. Among VUS, EVE scores adopted a binomial distribution with peak frequency of scores occurring between 0.2–0.3 and 0.8–0.9, but 28% of variants had scores in an intermediate range between 0.3 and 0.7 (supplemental Fig. S6B). Selecting VUS with uncertainty <0.6 removed all variants with EVE scores within the intermediate range. Decreasing the uncertainty threshold to <0.3 further selected for variants with scores only between 0.0 and 0.1 or between 0.9 and 1.0. ESM does not produce uncertainty or confidence measures with scores.

AF2 structures had an associated per-residue pLDDT score that reflected model confidence. Since |ΔΔG| values were explicitly calculated from the structure, model confidence was a more relevant measure of uncertainty compared to variance from ΔΔG calculations. Increasing the per-residue pLDDT threshold past 90, which is associated with high model confidence, decreased the number variants by 40% for both structures. However, AUC for structure 1 decreased from 0.83 to 0.801 and for structure 2 from 0.863 to 0.859 (supplemental Fig. S6C, D).

### Burden testing shows strong association between EVE and ESM scores and clinical phenotypes

**Fig. 4:**
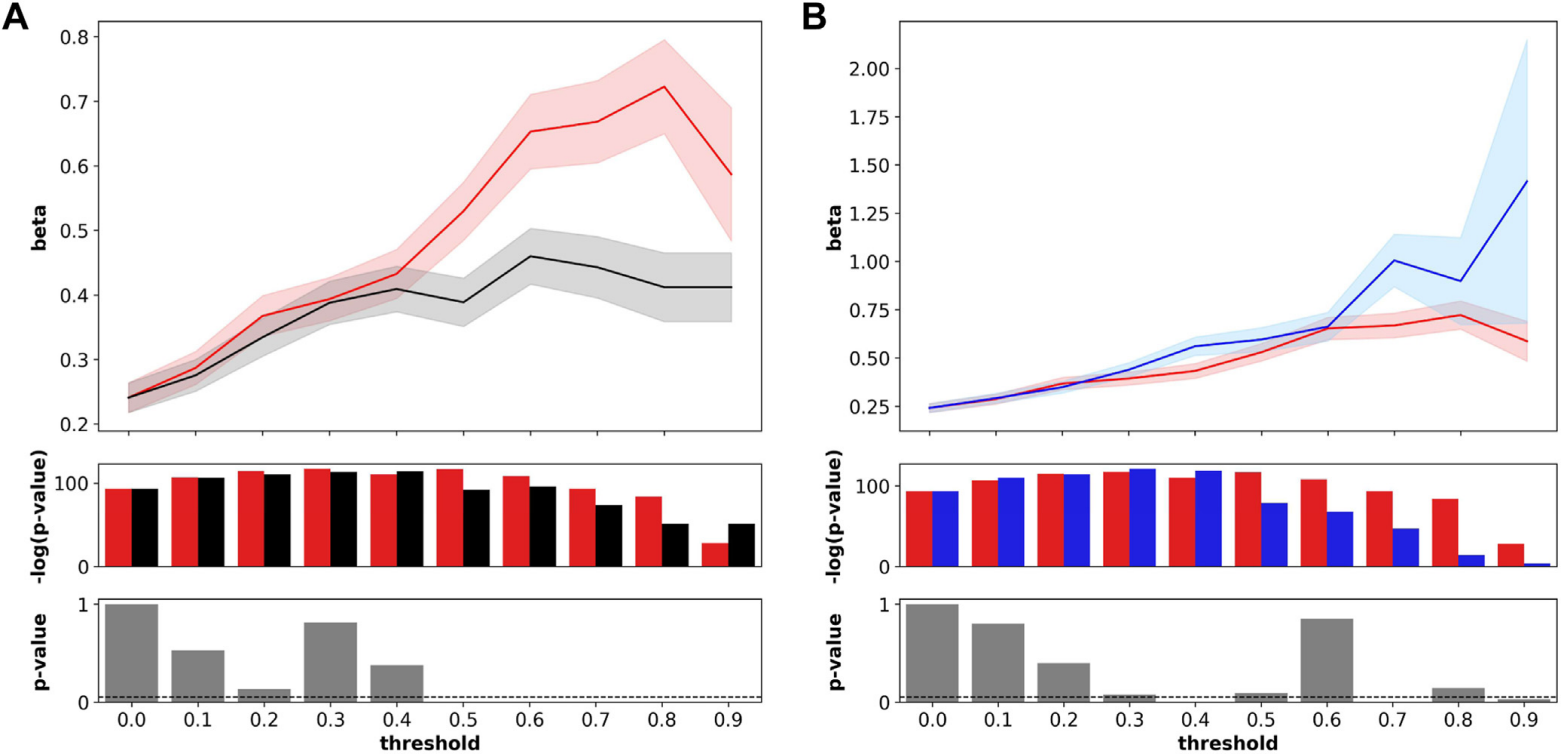
**Association between variants and serumLDL-Cby prediction score.** Associations were calculated between EVE (black) and (A) Polyphen-2 (red) and (B) ESM (blue). Scores were normalized as a percentile rank and the threshold included all normalized scores greater than or equal to it. (*Top Panel)* Association between LDL and prediction score thresholds were measured as effect sizes in standard deviations (beta). (*Middle Panel*) Significance of association is quantified as a negative log *P*-value. (*Bottom Panel) P*-values were calculated for difference between scores and the dashed lined marks the 0.05 significance threshold.

We tested for associations between EVE, ESM, and Polyphen-2 scores with LDL-C. For comparison, scores were normalized by percentile ranks based on an empirical cumulative distribution function calculated from all possible shared variants between the three scores. Variants were grouped based on their prediction scores, such that variants with scores greater than or equal to the threshold were labeled as pathogenic. Since defining a clear threshold proved challenging, we conducted tests across a spectrum of thresholds ranging from 0.0 to 0.9. This helped us identify a threshold that produced significant differences between scores. We restricted the analysis to missense variants within exons for a total of 626 unique variants. LDL-C values were inverse normal transformed, and the degree of association was quantified as standard deviation from mean (55). The associations between LDL-C and EVE and Polphen-2 were compared (Fig. 4A). Effect sizes were significantly higher for EVE than Polyphen-2 when thresholds were within the top 50th percentile of scores. In this window, effect sizes for Polyphen-2 increased from 0.39 sd. (95% CI 0.35–0.43) at a threshold of 0.5 to 0.41 sd. (95% CI 0.36–0.47) at a threshold of 0.9. For EVE, effect sizes range from 0.53 sd. (95% CI 0.48–0.57) at a threshold of 0.5 to a peak of 0.72 sd. (95% CI 0.65–0.80) at a threshold of 0.8. Next, the association between LDL-C and ESM was compared (Fig. 4B). ESM had significantly higher effect sizes than EVE at thresholds of 0.4, 0.7, and 0.9. For ESM, effect sizes range from 0.59 sd. (95% CI 0.53–0.66) at a threshold of 0.5 to a peak of 1.41 sd. (95% CI 0.68–2.14) at a threshold of 0.9.

**Fig. 5:**
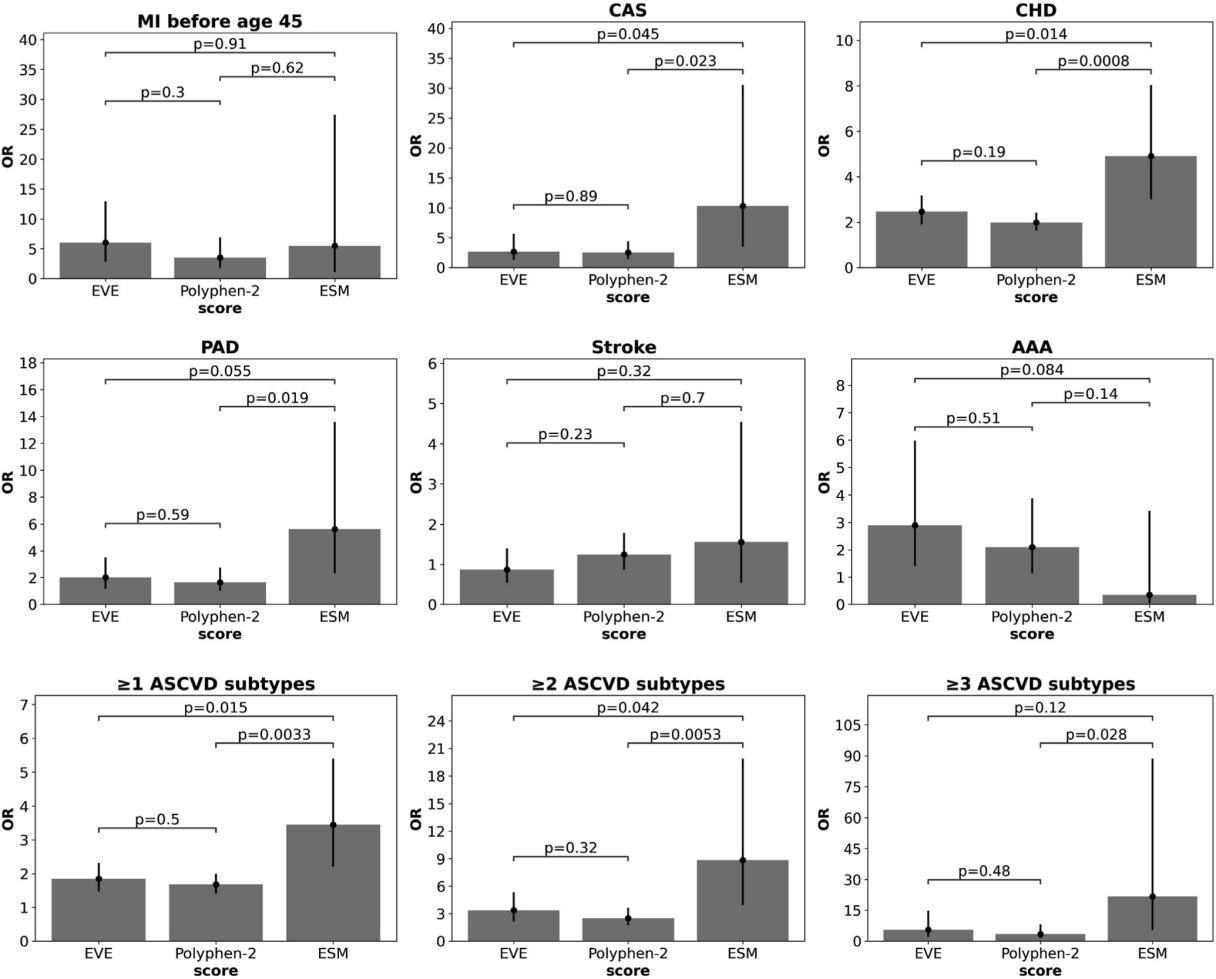
**Associations between variants andASCVDphenotypes by prediction score.** Variants with either EVE, Polyphen-2, or ESM scores in the top 30th percentile were associated with ASCVD diagnosis, calculated as an ORs. Differences between ORs were assessed using a two-sample independent *t* test with *P*-values displayed. AAA, abdominal aortic aneurysm; CAS, carotid artery stenosis; CHD, coronary heart disease; PAD, peripheral artery disease.

We next assessed the association between EVE, ESM, and Polyphen-2 scores and ASCVD phenotypes including carotid artery stenosis, coronary heart disease, peripheral artery disease, stroke, abdominal aortic aneurysm, and early MI (Fig. 5). We calculated an OR for each phenotype using the previously observed threshold of 0.7, which showed significant differences between Polyphen-2, EVE, and ESM. Generally, there were no significant differences between EVE and Polyphen-2 for all ASCVD phenotypes. Among individual phenotypes, ESM had significantly higher ORs for carotid artery stenosis and coronary heart disease. ESM had higher OR for peripheral artery disease compared to EVE and Polyphen-2, which was significant for Polyphen-2 but was slightly above a *P*-value of 0.05 when compared to EVE. However, in aggregate for at least one ASCVD diagnosis, ESM (OR 3.45, 95% CI 2.2–5.4) had a significantly higher OR compared to EVE (OR 1.85, 95% CI 1.47–2.32) and Polyphen-2 (OR 1.68, 95% CI 1.41–1.99). All three methods were associated with higher OR for two or more ASCVD diagnosis, but ESM (8.85, 95% CI 3.94–19.88) was significantly higher compared to EVE (3.36, 95% CI 2.11–5.34) and Polyphen-2 (2.49, 95% CI 1.72–3.61). Further filtering for cases with three or more ASCVD diagnosis shows ESM (21.7, 95% CI 5.31–88.59) to have a higher OR compared to EVE (5.48, 95% CI 2.03–14.8) that is not significant but is significantly higher compared to Polyphen-2 (3.39, 95% CI 1.42–8.08).

## Discussion

The Clinical Genome Resource variant curation interface supports the application of evidence criteria to classify variants based on the American College of Medical Genetics and Genomics and Association for Molecular Pathology guidelines (3). The annotation process combines clinical, genetic, population, and functional evidence with expert review to classify variants. As new variants are identified, curation continues to be a challenge, and novel approaches are needed to enhance variant classification. Our results suggest that deep generative models such as ESM and EVE have the potential to improve variant annotation.

ESM and EVE were remarkably competitive in their ability predict variant effects across structurally diverse domains in ClinVar when compared to previously established machine learning methods that have been directly trained from variant classification. Within ClinVar, EVE and Polyphen-2 performed the best. However, EVE and ESM had the strongest correlation with LDL uptake measured in experimental assays, while Polyphen-2 was among the weakest. The improved performance of EVE and ESM are remarkable considering they did not require supervised learning. Our results demonstrate the power of deep learning models which use a complex series of nonlinear correlations to encode epistatic interactions needed for protein function prediction, within their hidden space. Moreover, these results highlight that the relevant epistatic interactions needed for protein function prediction can be successfully learned from the evolutionary history of natural sequences.

Large population studies such as the UKB allow us to directly test association between risk scores and clinical phenotypes. Both ESM and EVE showed a greater correlation with serum LDL-C compared to Polyphen-2; however, higher ESM scores were associated with a more extreme LDL-C levels. The association with ASCVD diagnosis in the UKB was not significantly different between individuals with variants labeled pathogenic by EVE scores compared to Polyphen-2. When annotating variants using ESM, we observed a higher OR for carotid artery stenosis, coronary heart disease, and peripheral artery disease. Compared to EVE and Polyphen-2, ESM was also able to select for variants with a higher OR for more extreme cardiovascular phenotypes such as individuals with multiple ASCVD diagnosis. Our results suggest ESM is better than EVE for identifying variants with extreme phenotypes. These results also demonstrate that ClinVar classifications are not reliable for comparing performance between models.

One might expect that EVE would offer better predictions in general compared to ESM. This is because EVE relies on a protein-specific MSA for training which may produce more accurate representations of a protein compared to a generalist approach from ESM which trains over a global dataset of unrelated protein sequences that are also not aligned. Previous work has shown that the hidden representations of protein families between transformers trained on a universal corpus of sequences and VAEs trained on protein-specific sequences have a similar ability to cluster sequences by taxonomy (56). Within the hidden space, distances between sequences correlate with their evolutionary distance and representations of single variant sequences are clustered near to their WT sequence (19). However, the hidden spaces of ESM and EVE showed different distributions between benign and pathogenic variants in ESM that were not present in EVE. It is possible that this may be due to a more complex landscape within the VAE hidden space, which when reduced to two dimensions does show significantly different variant distributions by score. Another possibility is that ESM benefits from using a large and diverse dataset for training, which was shown in its own development to improve predictions (21). It is possible that while EVE may be capturing phylogenetic relations, it may suffer from a lack of diversity in sequences to predict smaller single amino acid changes. Moreover, the ELBO training function in EVE estimates the probability for an entire sequence, whereas the masked language model training object is explicitly designed to determine a conditional probability distribution for a given position and may be more suitable for transfer learning with variant effect prediction. The difference in likelihood estimation is reflected in the broader distribution of scores observed in ESM and may suggest EVE underestimates pathogenicity in variants with more extreme effects.

Using AF2 structures classified variant pathogenicity poorly compared to existing methods. The lower performance may in part be attributable to inaccurate prediction of ΔΔG values due to limitations of the model (57) and underlying noise in experimental data used to train model parameters (58). While both AF2 models yield an AUC of 0.86, this is a modest improvement to BLOSUM62, which is a naïve method utilizing only a crude quantification for differences in amino acid chemistry. Unlike EVE, we did not observe improved predictive performance with increasing model confidence. This is consistent with prior observations showing binary thresholds for functional prediction exclude a large number of functionally disruptive variants that have a neutral energetic cost (59).

Only two conformations were predicted by AF2 for LDLR which ultimately underscore a significant drawback in using AF2 for interpreting variant effects. While the algorithm relies on pattern recognition to generate structure, it does not incorporate knowledge of the actual folding process (60). Pathogenic effects from variants may affect a folding intermediate state or change protein dynamics that are important for allosteric interactions (61). In the case of LDLR, an open and closed conformation is observed based on pH and required for binding and releasing of its lipoprotein cargo. Both structures 1 and 2 appear to be variations of a closed conformation seen in acidic pH. However, the latter structure is not experimentally observed, demonstrating new interactions between LA repeats 2–6 and the beta-propeller domain that is produced by a hinge between LA repeats 4 and 5. These results corroborate prior observations showing inaccurate predictions in proteins with structurally heterogenous conformations from fold switching (62). AF2 likely selected the closed conformation as the most probable, and a second hinge point was developed to include additional interactions predicted from the MSA as opposed to further searching the conformational space for alternatives. AF2’s inability to accurately reproduce a heterogenous conformational ensemble for LDLR precludes its use in variant prediction and may also lead to errors in inferring mechanism of disease.

Study limitations include the fact that ESM, EVE, and AF2 models for LDLR were generated using default hyperparameters. In all models, hyperparameters were tuned to apply broadly across the whole human proteome. However, it is conceivable that models may be adjusted to better represent specific proteins. In the case of AF2 and ESM, this is not practical since accurate representations require training across a global set of protein sequences. Moreover, retraining weights is nontrivial, requiring sufficient computational resources. The EVE model is more convenient to retrain since it requires less data for training and is a smaller model. We performed a limited survey of different hyperparameters for generating a MSA or training EVE but were only able to marginally improve AUCs in ClinVar compared to the default values (data not shown). As a result, we did not pursue further optimization of EVE’s hyperparameters. A second limitation is a lack of high throughput experimental evidence such as multiplex assays of variant effects which measure a more significant fraction of possible variants and can better correlate EVE and ESM models with protein function and fitness. As mentioned, experimental assays for LDLR are difficult to automate, and the hope is that newer models such as ESM may serve as a substitute. Our study compares ESM and EVE with only a limited experimental dataset and would benefit from further comparison with high throughput functional assays. Recent high throughput methods to measure LDL-C uptake may provide an expanded dataset (63). Moreover, combining in silico and experimental models may result in improved predictive performance (64).

## Conclusion

We demonstrate that both ESM and EVE robustly classify variants across structural domains in LDLR. These models are significantly associated with experimental assays for LDLR function and cardiovascular phenotypes in the UKB, moreso than traditional prediction tools. Between EVE and ESM, ESM identified variants with more extreme clinical phenotypes consistent with higher serum LDL-C and multiple ASCVD diagnoses. In contrast, single structures generated from AF2 were unable to effectively model variant pathogenicity. Our results suggest that deep generative models such as ESM and EVE can improve accuracy of variant pathogenicity prediction.

## Data availability

Source code and parameters were downloaded from publicly available repositories: ESM (https://github.com/facebookresearch/esm), EVE (https://github.com/OATML/EVE), and AlphaFold 2 (https://github.com/deepmind/alphafold). ClinVar labels were downloaded from a public archive (https://www.ncbi.nlm.nih.gov/clinvar/). Experimental data from LDLR uptake assay was provided as a supplement from Thormaehlen *et al.* (8). The UK Biobank 500k exome sequencing dataset was provided on DNAnexus as described.

## Supplemental data

This article contains supplemental data.

## Conflict of interest

The authors declare that they have no conflicts of interest with the contents of this article.
