# Supplementary material for: Deep generative models of LDLR protein structure to predict variant pathogenicity
Source: J Lipid Res. 2023 Oct 11;64(12):100455. doi: 10.1016/j.jlr.2023.100455 (PMC10696256; doi:10.1016/j.jlr.2023.100455)
Supplement: Supplemental Figures S1–S6 [file mmc1.docx]

**Supplementary Figures**


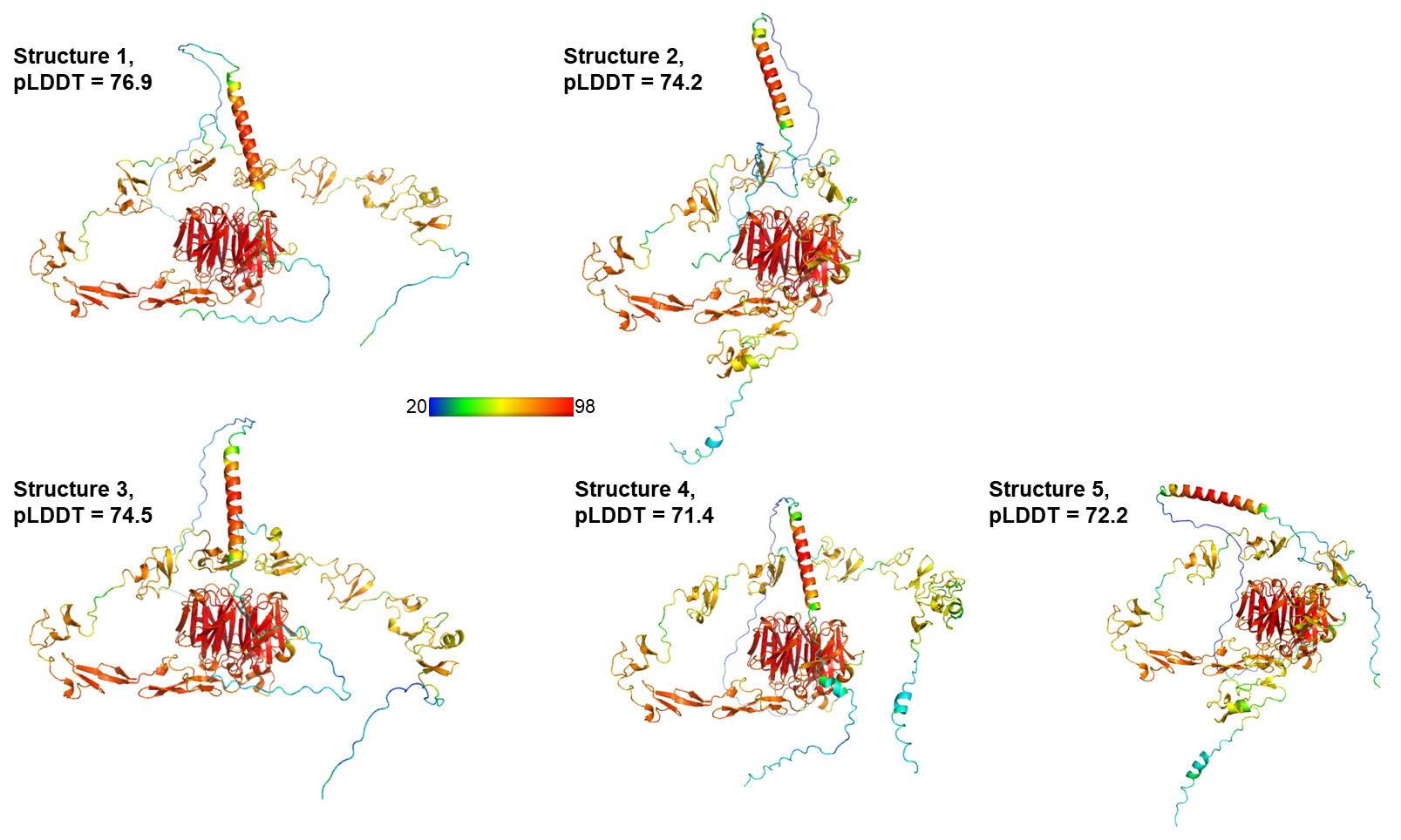


**Suppl. Figure 1.**  Five generated structures from AF2 for the complete LDLR protein sequence. Structures are labelled with a global pLDDT score and colorized by per-residue pLDDT score, colorbar represents range of scores.


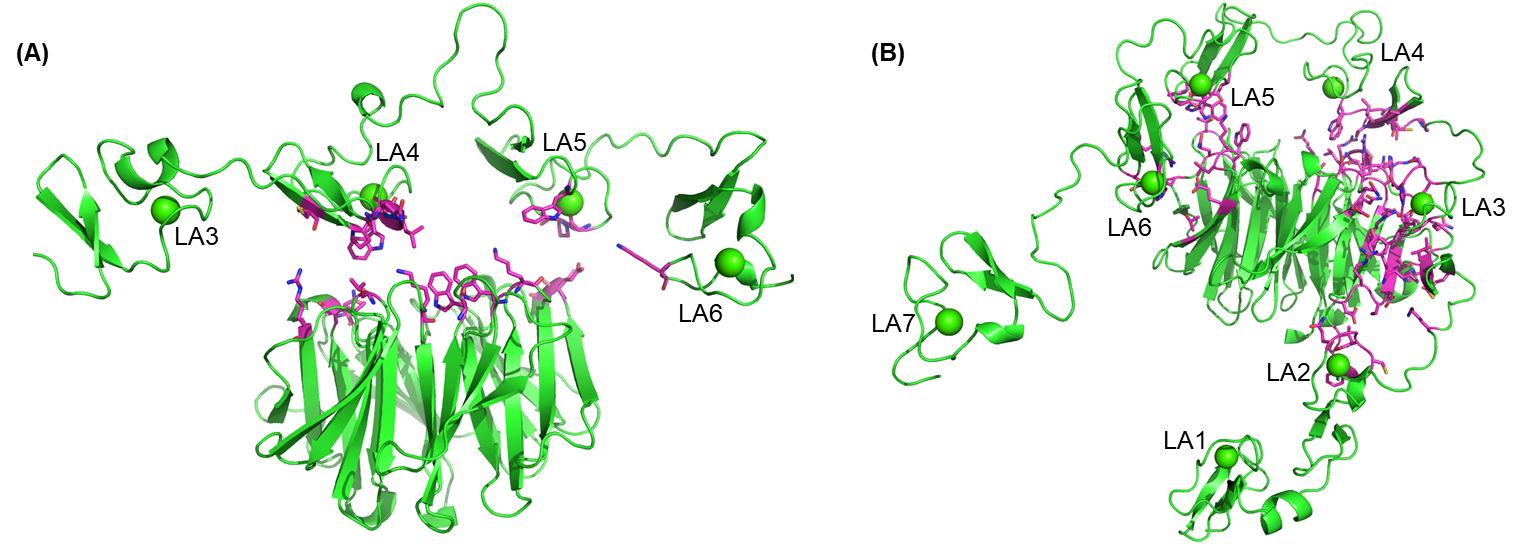


**Suppl. Figure 2.** Interface residues identified between LA repeats and the beta-propeller domains in AF2 preducted structures **(A)** 1 and **(B)** 2. Interacting residues were identified using a distance cutoff of 1.0 Å.


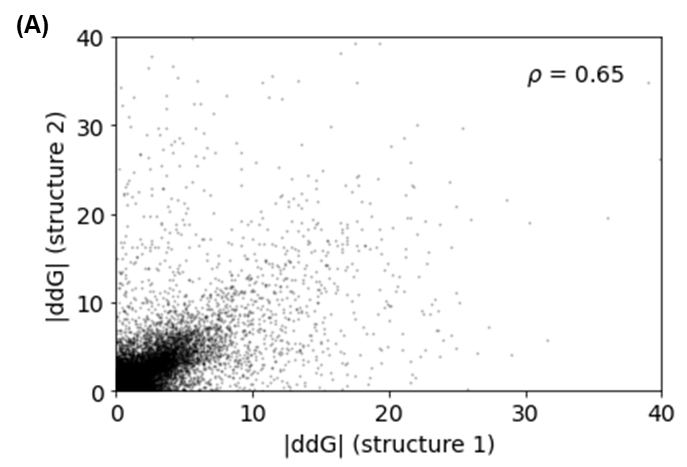


**Suppl. Figure 3.** Correlation between |ddG| values derived from variants modeled on AF2 predicted structures 1 and 2. Spearmen coefficient (ρ) labelled on plot.


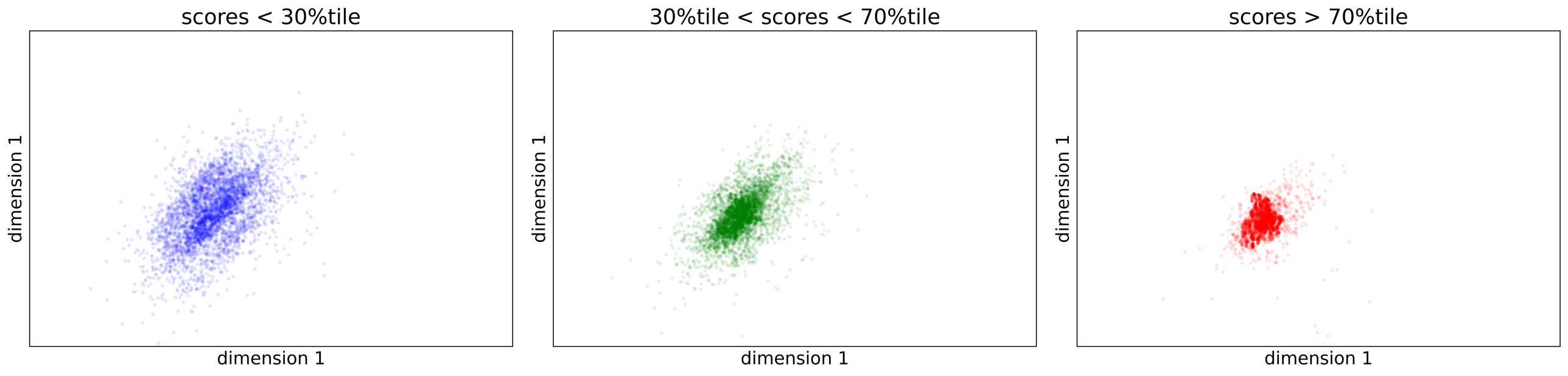


**Suppl. Figure 4. EVE with a two-dimensional latent space representation.** An EVE model was trained with a two dimensional latent space for visualization. The MSA and other hyperparameters were unchanged from the original model. Panels contain variants that are categorized by their percentile ranks.


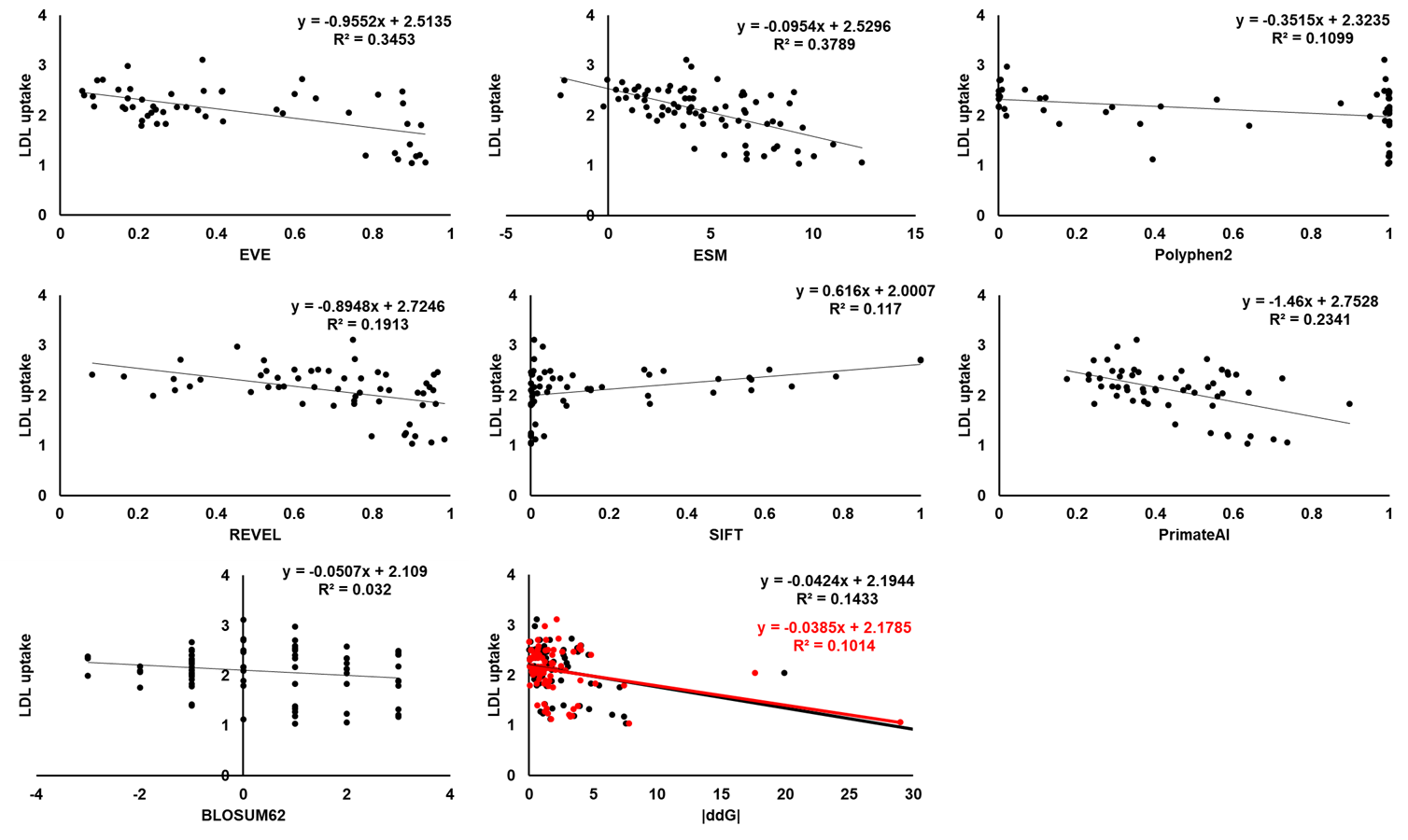


**Suppl. Figure 5.** LDLR function was quantified from a previously published experimental assay measuring cellular LDL uptake. Pathogenic variants had less LDL uptake than benign. Scores from EVE, ESM, Polyphen2, REVEL, SIFT4G, Primate AI, BLOSUM62 and |ddG| values from AF2 structures 1 (black) and 2 (red) were compared.


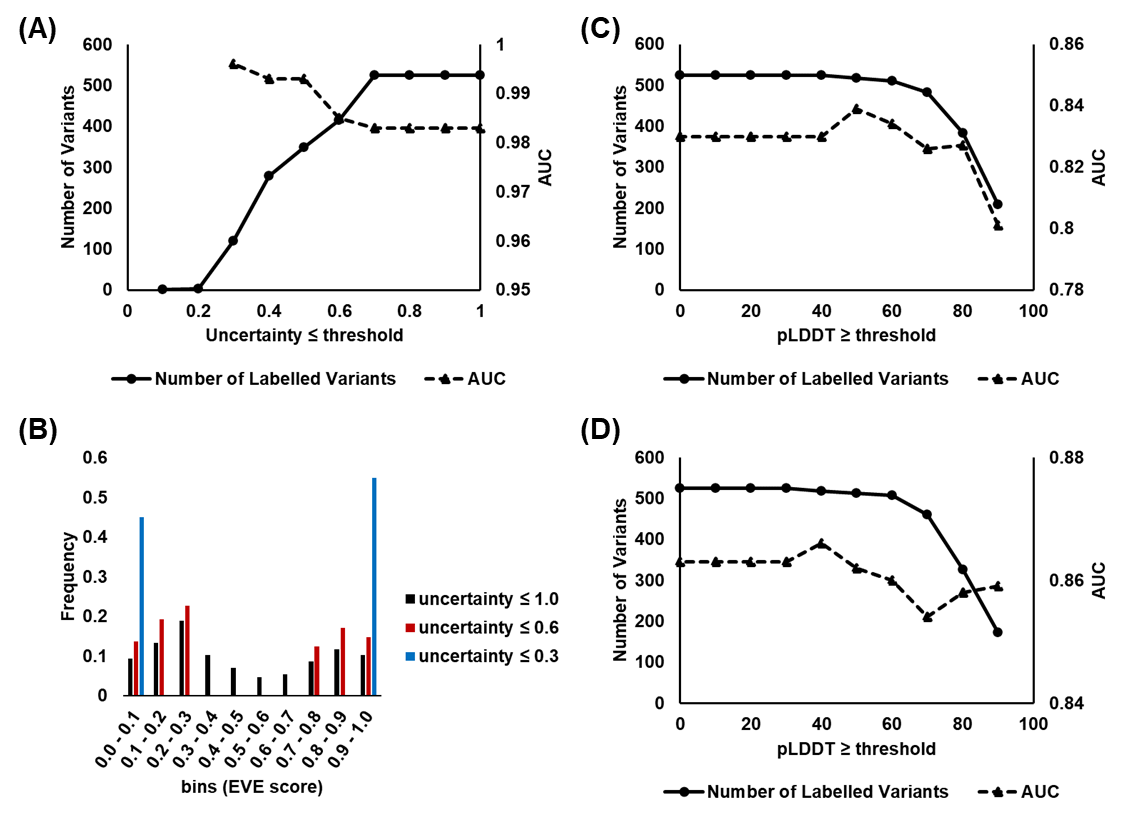


**Suppl. Figure 6.** EVE scores for a given variant are accompanied by an uncertainty metric where higher values represent greater predictive entropy. **(A)** AUC is calculated from EVE scores based on an uncertainty threshold (right axis). The number of variants included is also shown (left axis). **(B)** Distribution of VUS by EVE score based on uncertainty threshold. For AF2 structures, per residue pLDDT quantifies predictive confidence and higher values indicates greater model confidence. AUCs are calculated from |ddG| scores in **(C)** structure 1 and **(D)** 2 based on a pLDDT threshold (right axis). The number of variants included is also shown (left axis).
